# Stigma, psychosocial and economic effects of yaws in the Philippines: an exploratory, qualitative study

**DOI:** 10.1186/s41182-022-00433-4

**Published:** 2022-07-06

**Authors:** Belen Lardizabal Dofitas, Sherjan P. Kalim, Camille B. Toledo, Jan Hendrik Richardus

**Affiliations:** 1 Department of Dermatology, College of Medicine, University of the Philippines Manila, Metro Manila, Philippines; 2 Department of Pathology, Cotabato Regional and Medical Center, Sinsuat Ave., Cotabato City, Philippines; 3 Institute of Psychiatry and Behavioral Medicine, Southern Philippines Medical Center, Davao City, Philippines; 4 Department of Public Health, Erasmus MC, University Medical Center Rotterdam, Rotterdam, The Netherlands

**Keywords:** Yaws, Psychosocial, Stigma, Philippines

## Abstract

**Background:**

Yaws is a chronic, non-venereal, highly contagious skin and bone infection affecting children living in impoverished, remote communities and caused by *Treponema pallidum* subspecie *pertenue*. Social stigma and economic losses due to yaws have been reported anecdotally in the Southern Philippines but have not been well-documented.

**Objective:**

To describe and compare the psychological, social, and economic effects of yaws from the perspective of patients, contacts, and key informants in two areas of the Philippines.

**Materials and methods:**

Yaws and contacts were identified through clinicoseroprevalence surveys conducted in the Liguasan Marsh area, Mindanao, Southern Philippines in 2017 and among the Aetas, an indigenous people community in Quezon province, Luzon region in 2020. Skin examinations and serologic tests confirmed the diagnosis of active, latent, or past yaws among the children and adults. Trained health personnel conducted in-depth interviews of those affected by yaws and their guardians, household contacts, and key informants, such as health workers regarding their perceptions, feelings, health-seeking behaviors, and effects of yaws on their lives.

**Results:**

A total of 26 participants were interviewed: 17 from Mindanao and 9 from Luzon. Aside from the physical discomforts and embarrassment, yaws was considered stigmatizing in Mindanao, because positive non-treponemal tests or treponemal antibody tests were associated with syphilis and promiscuity. These have led to loss of employment and income opportunities for adults with latent or past yaws. In contrast, the Aetas of Luzon did not perceive yaws as stigmatizing, because it was a common skin problem. Plantar yaws interfered with the Aeta’s gold panning livelihood due to the pain of wounds.

**Conclusions:**

Yaws is not merely a chronic skin and bone disease. It can lead to significant psychosocial and economic problems as well. Yaws is a generally forgotten disease in the Philippines. There is no yaws surveillance and control program. Treatments are not readily available for the populations affected, thus perpetuating the infection and negative effects.

**Significance of study:**

This is the first study to document the psychosocial and economic effects of yaws among Filipinos. Information campaigns about yaws and a yaws control program are needed to reduce stigma and discrimination.

## Introduction

The skin is an important social and psychological interface between the individual and the environment. Even minimal flaws in the overall appearance of the skin can have a profound effect on the ego and, in turn, on an individual’s body image [1]. According to Gupta [2], approximately 30% of dermatological patients have associated psychiatric comorbidity as well as psychosocial stress. Disfiguring cutaneous conditions can result in social disapproval and increased self-consciousness which can result in problems in the workplace, social withdrawal, academic underachievement, and serious psychologic and body image problems especially when the dermatologic disorder occurred during critical periods in development, such as the adolescent period. This is significant, as social stigma can occur even in dermatologic disorders that do not have a primary psychosomatic component.

The Neglected Tropical Diseases (NTD), especially those with visible signs and deformities, have elicited fear and ostracism. Social stigma comprises the “hidden burden” of NTDs [3]. NTDs with chronic sequelae, such as Buruli ulcer and Leprosy, have been shown to have a greater association to developing psychiatric conditions, such as anxiety disorder and depression [4]. This is due to the physical impairments and disfiguring effect that go beyond the acute course which could subject an individual to social and self-stigma [5, 6]. This, in turn, affects an individual’s social interactions and self-esteem [5]. Anxiety and depression could also affect attendance to school and employment of affected individuals which could trap communities in endless cycles of poverty and cost developing economies billions of dollars every year [7, 8].

Yaws is a skin NTD characterized by yellow-crusted papules and nodules, ulcerations, and bony deformities due to *Treponema pallidum pertenue*, a spirochete very closely related to *Treponema pallidum pallidum*, which is the etiologic agent of syphilis. Yaws is found in humid, tropical countries and mainly affects children below 15 years of age [9].

Active yaws refers to the infectious stage (primary and secondary yaws), where skin lesions containing the bacteria can be transmitted by direct skin contact. A tertiary stage with destructive bone disease can develop when yaws remains untreated. In between stages, yaws enters a latent stage with no obvious skin lesions, often in adulthood. Yaws can be treated with penicillin or azithromycin and renders the patient non-infectious (past/treated yaws) [10]. Yaws may be stigmatizing due to disfiguring bony complications and skin ulcerations and has caused absenteeism among schoolchildren [11]. Aside from the physical problems and disabilities caused by NTDs, such as yaws, the psychosocial burden is also important to measure and mitigate to reduce human suffering.

We did an electronic search for published literature using the search terms “yaws,” “stigma”, “psychosocial”, and “mental health” (PubMed, Google Scholar, InfoNTD) up to May 25, 2022, but did not find research specific for yaws. Studies on yaws are mostly concentrated on its identification and eradication [12]. Literature on yaws interventions have been primarily pharmacologic.

The psychological and psychosocial impact of NTDs as well as interventions focused on these aspects have recently gained recognition. However, published literature are still few and lacking. In a review by Bailey et al. in 2018 [5], there were only three NTDs with psychological intervention studies (cutaneous Leishmaniasis, snake bite, leprosy) [5, 13–15] and three with social intervention studies (Buruli Ulcer, Leprosy, Mycetoma) [5, 16–18].

One author of the current paper (BLD) first heard anecdotal reports of the social stigma and discrimination associated with yaws in the year 2000, while she was conducting a health-seeking behavior study and skin survey in the Liguasan Marsh communities of Mindanao, Southern Philippines. The stigma and discrimination associated with yaws may vary across the disease condition and the culture in affected communities. According to Hotez [19], “there is not a ‘‘one size fits all’’ interdisciplinary approach to reduce stigma.” Social science research on yaws is needed to strengthen approaches in the control and eventual eradication of this disease.

In the Philippines, yaws was last reported by the health authorities in 1973, surveillance ceased, and yaws was thought to have been eradicated. However, confirmed current and past cases were detected in Southern Philippines in 2017, making the Philippines the 14th yaws-endemic country in the world [20].

This report aims to present the results of exploratory qualitative studies that were conducted together with focused clinicoseroprevalence and case detection surveys done in 2017 and 2020 in two different parts of the country. This report presents the physical, psychosocial, and economic effects of yaws among confirmed patients, household members, and the community at large.

## Methods

### Ethics statement

The two yaws study proposals were registered with and approved by the Technical Review Committee of the Philippine Council for Health Research and Development (PCHRD) (PHRR 191212-002355; PHRR 10803-001361). Ethical approval for the 2017 yaws study was granted by the St. Cabrini Medical Center–Asian Eye Institute Ethics Review Committee (SCMC–AEI ERC No. 2016-022). The ethical approval of the 2020 yaws study proposal was granted by the Single Joint Research Ethics Board (SJREB 2018–24) and the University of the Philippines Manila Research Ethics Board (UPMREB 2018-53401). Written informed consent to participate in the study were secured from the parents or guardians of minors and from adult participants. Written informed assent were secured from minors aged 6–18 years of age.

### Detection of yaws

The data on the psychosocial effects of yaws were collected during two exploratory yaws epidemiological studies conducted in the Philippines by Dofitas, et.al.

Yaws I Study: The first yaws study was conducted from February to May 2017 in order to confirm the presence of yaws in the Southern Philippines. The Department of Health commissioned a clinico-seroprevalence and case detection survey in three provinces of Mindanao, Southern Philippines, where yaws reportedly continued to exist: Maguindanao, Cotabato, and Sultan Kudarat. Three municipalities per province situated in Liguasan Marsh were purposively chosen based on previous yaws reports. School-based and community case detection surveys were conducted in one randomly selected public elementary school per selected municipality, totaling nine schools. Case detection was also conducted among household contacts of students and community members.

Yaws II Study: In 2019–2020, the Department of Health commissioned a second case detection study to search for yaws in the remaining major island groups of the country, Luzon and Visayas. There were five purposively selected remote communities that were historically endemic for yaws. Selected villages were in the provinces of Bataan, Rizal, and Quezon in Luzon, and Samar and Leyte provinces in the Visayas. Two study sites (Rizal and Quezon) were selected also, because there were anecdotal reports of yaws among the indigenous people residing there.

The details of the first confirmed active, latent, and past yaws patients in the Philippines since the 1970s have been published in 2020 [20]. The 2017 clinico-seroprevalence survey methodology and findings are published in a separate article [21].

### Sample size

The number of participants were dependent on the presence and number of people affected by yaws that were detected per study site. We targeted 2 to 5 participants with yaws per study site and 2 to 5 participants who were considered risk-prone (i.e., household contacts of yaws patients). We planned to interview at least one key informant per study site with yaws.

### Selection of participants

The primary informants were purposively selected participants with yaws (active, latent, past) who were diagnosed during the case detection surveys. Soon after the yaws participants were detected, they were approached by field personnel and invited to participate in the in-depth interviews. For yaws patients who were minors, at least one parent was interviewed on the minor's behalf. Older children (aged 7–18 years) were also interviewed with their assent. Target key informants were health professionals and health workers who had encountered yaws.

### Qualitative study design

We used a rapid ethnographic assessment (REA) method consisting of exploratory qualitative interviews (i.e., key informant interviews and in-depth interviews) and observations of participants by field assistants to acquire initial insights of the cultural meanings and scripts attached to yaws.

Rapid ethnographic assessment is a pragmatic and participatory qualitative method used when there is a need for more information about a problem that very little is known about or when situations are poorly understood, as in the case of yaws and its psychosocial repercussions. REA is also useful when there is a need to reach hidden or vulnerable populations, because this method encourages the involvement of trusted local community members in data collection who, in our case, were the local health staff and village health workers. This method centers on indigenous or local knowledge and community perspectives [22].

Local terms for yaws were gathered by the investigator from interviews with residents of the study sites and from searching online for dictionary terms and historical documents on yaws in the Philippines and any psychosocial effect on those affected.

### Development of interview guides

Interview guides for in-depth interviews of yaws participants and key informants were adopted from the guides used in a health-seeking behavior study on Filipinos with common skin diseases conducted by the principal investigator [23]. The guide questions were translated into the local languages of the study sites (Filipino, Hiligaynon, Visayan, Waray).

The translators were native speakers of the local language and fluent English speakers. There was one translator per step (ex. forward and back translation). The field supervisor checked the translation and approved the forward and back translation.

For face validity pre-testing of the interview questions, 2 to 3 native speakers of the local language were asked to read the translated forms, if possible, or the field assistant read the questions to the participants. The pre-test participants were asked for their feedback and suggestions on clarity and ease of comprehension. In general, the translations were rated as clear and easy to understand. All translated questions were approved by the ethics review boards. Participants of the pre-testing were not participants of the yaws studies.

### Study procedure

#### In-depth interviews

Local health staff underwent a brief training on the interview methods. They conducted semi-structured in-depth interviews among confirmed yaws participants (active, latent, and past yaws), their guardians, and household contacts, and key informant interviews of health workers and a local leader. They were encouraged to add probing and clarifying questions on various issues that related to the participants’ experiences in having the disease. The in-depth interview, which lasted for about an hour, was conducted in a venue recommended by the participant. The interviewers spoke the local language, were not residents of the sample area but worked there as health staff.

The list of topics for discussion included:

1. Personal knowledge, beliefs and attitudes including social stigma
2. Life events attributed to the disease
3. Current behaviors and behavioral predispositions about managing the disease
4. Socioeconomic effects of yaws

The separate interviews done with the household contacts allowed the interviewer to make general observations about the contacts’ own beliefs and attitudes as well as health-seeking behaviors or practices.

#### Key informant interviews

The following information were asked:

1. Personal knowledge about people affected by yaws (year when yaws was first encountered, number of people affected, manifestations, how diagnosis was established, management).
2. Beliefs and attitudes about yaws.
3. Socioeconomic effects of yaws on the patient, family, community.

The field notes and the verbatim transcriptions of the interviews and narratives were filed using an analytic memo system. This involved the organization of data based on the concepts and themes that emerged from the data. The texts were read, significant words, phrases, sentences were highlighted, and key ideas that emerged were written and reflected upon.

## Results

**Fig. 1:**
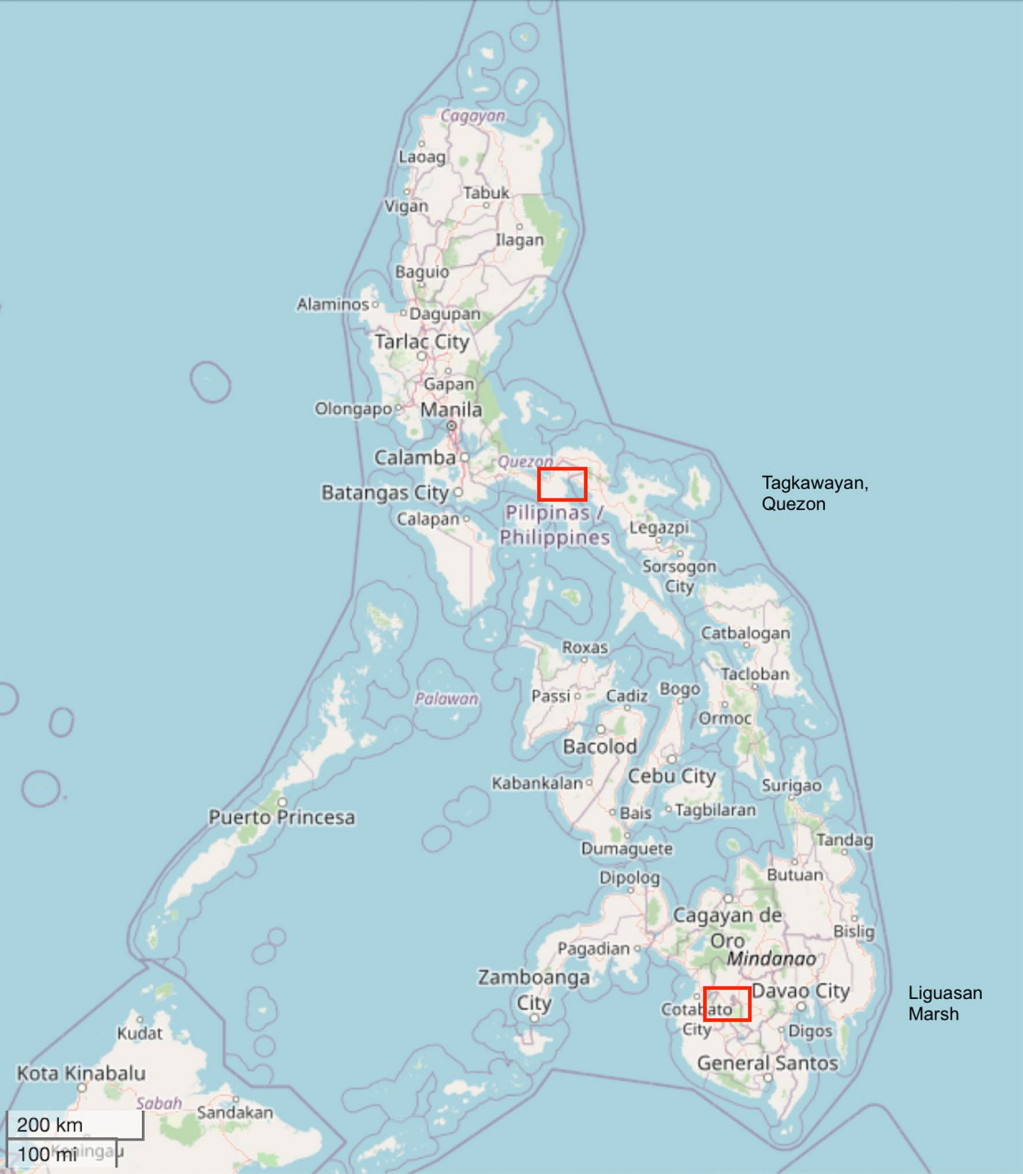
General location of communities affected by yaws in the Philippines. (Contains information from OpenStreetMap and OpenStreetMap Foundation, which is made available under the Open Database License) [24]

This qualitative study was conducted in the Northern and Southern Philippines, where yaws was detected (Fig. 1). A total of 3018 children and adults were examined for any skin disease during the Yaws I and Yaws II studies (*N* = 2779 and *N* = 239, respectively). A total of 33 people affected by yaws [active (*n* = 9), latent (*n* = 10), past yaws (*n* = 14)] were detected during the two yaws studies, of which 18 (54%) participated in the qualitative phase of the studies. The remainder had either not given consent or were not residing in the study site anymore when the interviews were conducted. There were key informants only from the Mindanao, Southern Philippines study sites (*n* = 8) of the first yaws study because there were no eligible health workers who had previously encountered yaws in the Quezon province, Luzon study site.

The findings of the two yaws-endemic communities shall be described separately.

### Characteristics of participants

We included a total of 26 participants: 18 yaws participants, guardians, and household contacts and 8 key informants (Table 1).

**Table 1:** Participants of in-depth interviews and key informant interviews

		Yaws I Study (Southern Philippines)				Yaws II Study (Luzon/Visayas)	Total
		Datu Piang, Maguindanao	Columbio, Sultan Kudarat	Kabuntalan, Maguindanao	Tulunan, Cotabato	Tagkawayan, Quezon	
Yaws cases and contacts							
Mean age (SD) (years)	36.7 (14.0)					31.3 (11.8)	
Range	7–58					22–52	
Female	5					5	
Male	3					4	
Not specified	1						
Active yaws			1		1	1	3
Latent yaws		1		1	1	1	4
Past yaws						6	6
Household contact		1		2	1	1	5
Total		2	1	3	3	9	18
Key informants							
Mean age (SD) (years)	43.9 (13.8)						
Range	28–60						
Female	6						
Male	1						
Not specified	1						
Doctor		1			1		2
Village health worker				1	1		2
Teacher			1				1
Nurse			1		1		2
Midwife					1		1
Total		1	2	1	4	0	8

In the Southern Philippines sites, there was a total of 17 participants from the 3 provinces. Five yaws participants (2 active and 3 latent) and three guardians/household members had in-depth interviews. There were three males and 5 females (one participant’s gender was not specified) with a mean age was 36.7 years (SD 14.0; range 7–58).

Eight health personnel and long-time members of the community (head teacher) were key informants. Six females and one male (one participant’s gender was not specified) with a mean age was 43.9 years (SD 13.8; range 28–60 years) were participants.

In the Yaws II study, there were nine participants from the Tagcawayan, Quezon province study site for the in-depth interviews: active yaws (*n* = 1), latent yaws (*n* = 1), unaffected household contact (*n* = 1), and past yaws (*n* = 6). There were 5 females and 4 males, all adults with a mean age of 31.3 years (SD 11.8; range 22 to 52). There were no key informants among the local health care workers as they had never encountered yaws in the past and the Aeta community had just recently registered as residents in Sitio M.

In the other study sites, the study team members asked the local health workers and leaders if they had local terms for yaws or had seen yaws-like skin lesions in the past or present.

## Yaws I. Liguasan Marsh, Mindanao (Southern Philippines)

Liguasan Marsh is a centrally located, large catchment basin in Mindanao, found within three adjacent provinces: Maguindanao, Cotabato, and Sultan Kudarat. The communities living in Liguasan Marsh are generally impoverished and difficult to access due to remoteness and long-running internal conflicts between rebel and government forces. The population is predominantly Muslim.

### Personal knowledge, beliefs and attitudes including social stigma

#### Local terms for yaws

Participants were asked what their skin disease is called. Yaws was mainly referred to as “bakataw”, the Maguindanaoan term for yaws skin lesions. Others referred to it as “allergy” or “*makati”* (itchy). Health personnel reported that the local terms were “*bakukang*” (*n* = 4), “*bakataw*” (*n* = 3), “*kaluli*” (*n* = 2), “*burok*” (*n* = 1), and “*topak*” (*n* = 1).

Aside from “bakataw”, the following Maguindanaoan terms of yaws were found through interviews:

- “*Pamali*” = crab yaws; yaws papillomas and wounds on the soles of the feet.
- “*Bungkot*” = lesions when skin peels off due to crab yaws.
- “*Bakukang*” = ulcers due to yaws; a general term for deep wounds/ulcers on the skin that may have various etiologies.

The Visayan terms found in an online Cebuano-Visayan dictionary) [25] were “puku” and “tabukaw.” During the Spanish colonial era, yaws was referred to as “bubas” [26].

#### Duration of presence of yaws in the study sites

One government doctor who had worked in Maguindanao for around 35 years had seen several community members affected by yaws since the 1980s. She procured injectable penicillin and treated yaws patients she encountered or those who would seek treatment from her. The improvement and clearance of the yaws lesions was very quick. Over the years, she saw less and less yaws patients.

One of the health personnel had known about yaws in the community for over 15 years. Two of the health personnel had known about yaws for about 1 year, while three participants only found out about yaws recently through this study.

#### Areas where yaws has been seen

Health personnel reported having seen yaws in the following areas:

1. Datu Piang, Maguindanao
2. Dungos, Tulunan municipality, Cotabato province
3. Lutayan, Sultan Kudarat province
4. Sigayan, Lambayong, Sultan Kudarat province
5. Esperanza, Sultan Kudarat province
6. Coastline of Buluan Lake (between the provinces of Sultan Kudarat and Maguindanao)

One doctor from Maguindanao had seen several yaws patients, mainly pediatric, from 1984 up to the early part of the 2000 decade. She has been seeing less patients since then. She had made written reports to local health authorities about yaws patients in the past. However, there were no reports of yaws in the Department of Health Disease Surveillance Statistics. We requested reports of yaws from the Integrated Provincial Health Office of Maguindanao and other study sites but these were not found nor provided. For the other health personnel, around 6 pediatric yaws patients were seen, one young adult, and one elderly case.

#### Appearance and evolution of yaws lesions

Most of the patients and household members recall that the yaws lesions first appeared as small, firm but enlarging, itchy “bumps” or papules that the patients scratched until wounds or scaling/peeling appeared. Some skin lesions looked like boils or furuncles or had yellow discharges.

**Fig. 2:**
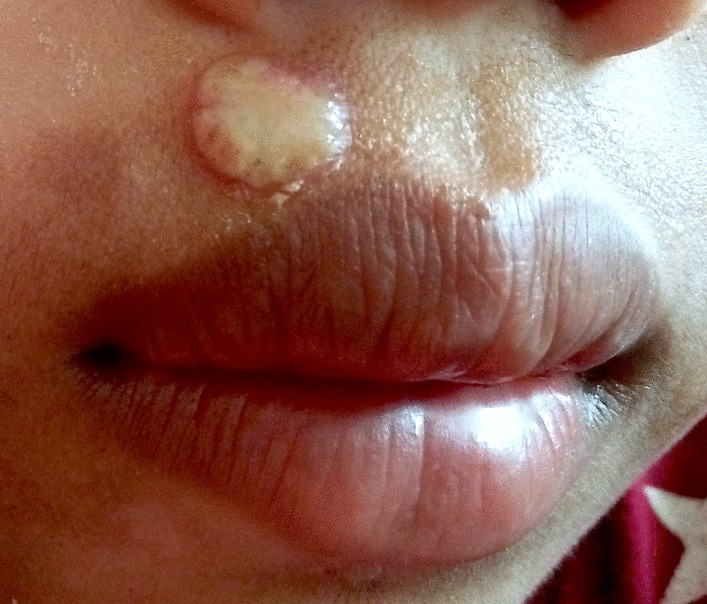
Yaws skin lesion: moist cauliflower-like papilloma (Photo credit: Dr. Camille Toledo)

Health personnel described yaws skin lesions as cauliflower-like with cheesy material or pus, erythematous lesions with a cheesy appearance, crusts with “juicy” pus underneath, and an unusual skin mass with whitish patches (Fig. 2). No bone lesions were noted.

One patient had severe bone pains as the skin lesions evolved, indicating bone inflammation due to yaws.

“*May tumubo na maliit…parang laman na mamula-mula o manilaw-nilaw hanggang sa pati mga buto ko ay nakararamdam na rin ng sakit*”

(Small things grew, like reddish or yellowish flesh until even my bones felt painful)

Two patients could not recall how the yaws lesions looked like because they were young when they had it.

#### Physical effects of yaws

According to key informants from Mindanao who were health workers, the yaws infection creates skin and cartilage deformities. Multiple scars make the skin look rough. The skin lesions have a fishy smell and they look unsightly and unhygienic. The yaws lesions are considered degrading, repulsive, and disgusting (“*kadiri*”). Some health workers observed that activities of daily living would not be impaired during early disease or in childhood. During the later stages of yaws, activities would be impaired, such as regular work due to the itching, pain of wounds on the soles, and the disruption in daily routine due to the time taken up to clean wounds. Activities would also be affected by the patients’ feelings of depression, isolation, and discrimination by others.

Yaws patients reported that they felt disturbed by the itchy skin lesions even during their sleep. One patient felt underlying bone pain.

### Life events attributed to the disease

Interviewers asked about knowledge or perceptions on the causes of yaws.

Patients/household (HH) contacts: Four of the patients/HH participants did not know the cause of yaws. The concept of yaws as a contagious disease was apparent in the responses of the other 5 participants.

Three participants attributed yaws to exposure to the water in the marsh when bathing or fishing, while the remainder associated it with lack of proper hygiene (ex. not washing body before bedtime). One participant replied that yaws was acquired from a neighbor, a playmate who also had yaws. One participant thought that the neglect of the parent led to the infection.

Health personnel: Poor sanitation and lack of hygiene were the most common causes cited by the participants. Three participants knew yaws was a bacterial infection caused by *Treponema pallidum* (*n* = 2) after they attended the yaws orientation. Yaws was thought to be transmitted by swimming in contaminated water (*n* = 2), skin-to-skin contact (*n* = 1), or by family heredity (*n* = 2).

When asked why they think yaws is present in their communities, 4 health personnel attributed yaws to the proximity to the marsh and remoteness of the Muslim communities. Poor sanitation, low level of education or ignorance, culture, poor socioeconomic status, and poor nutrition were also factors mentioned. Yaws is perceived to be communicable, curable, and maybe preventable.

### Current behaviors and behavioral predispositions about managing the disease

Health-seeking behaviors: Four participants reported that they did not do anything at all to treat the yaws lesions. Eight out of 9 participants did not have the yaws treated initially, because there was no health center or it was too far at that time (*n* = 4), the lesions resolved spontaneously (*n* = 2), or the patient treated him/her self (*n* = 2). One patient consulted at the health center and was given antibiotics.

Remedies and treatments: Four participants washed the skin lesions and used herbals, such as guava leaf decoction. Topical home remedies used were petroleum jelly, moisturizing soap (brand: Oilatum), and heat rash powder (brand: Fissan). One participant did not bathe the patient when there was fever.

Three participants eventually had the yaws treated in the health center. Two patients applied penicillin powder and one was given amoxicillin. The yaws lesions dried up.

Decision to seek treatment: Participants sought treatment because of their desire to get well, the disturbing itch, and because the skin lesions looked dirty.

Influences to seek treatment: The elders or older relatives (ex. uncle) or mother were the ones who advised treatment. Health workers (ex. Barangay health worker, midwife) also convinced participants to seek treatment. Only one participant sought treatment on his/her own.

#### Diagnostic tests, treatments, and health services available

Five health personnel said that there are no diagnostic tests for yaws available in their communities, while the Lambayong Rural Health Unit, Sultan Kudarat had tests available. Available treatments mentioned were azithromycin, “injections”, benzathine penicillin (ex. Penadur), povidone–iodine, and antibiotic ointment (ex. Fucidin). Good hygiene, medication, and regular follow-up have been advised to yaws patients.

Health services for yaws such as skin check-ups and health education are available in health centers. Information materials on yaws are currently available through the yaws study.

### Socioeconomic effects of yaws

Among the patients, 4 reported that yaws had no effect on their lives, since the patients were young and could still play and interact with others or they could lead normal lives. They felt no stigma due to yaws probably due to the low level of knowledge about yaws in the community. Five out of 9 yaws participants noted negative effects on their lives.

#### Psychosocial effects of Yaws

1. Stigma, shame, embarrassment due to positive VDRL, RPR, or treponemal antibody tests that are often assumed to be due to sexual transmission of syphilis
  Among the psychosocial effects of yaws, the stigma caused by a positive screening or confirmatory test for a Treponemal infection is the most significant. Screening test such as VDRL or RPR are routinely required for overseas employment applications. Although these tests are not only for screening for sexually transmitted infections (STIs), a positive screening test is immediately associated with the STI, venereal syphilis. A positive VDRL, RPR, or treponemal antibody test is, therefore, highly stigmatizing because of the association with promiscuity and fear of contagion.
  Shame and embarrassment were expressed by two adult patients who had positive VDRL results in the past and who were found to have latent yaws during this study.
  A 42-year-old woman from Kabuntalan, Maguindanao had yaws when she was 7 years and was left with dark scars on her legs and feet. She felt shame and stigma due to her positive VDRL test result. She was told by her friends that she acquired it through sexual intercourse with men and that made her blood “dirty”. In effect, she was also made to feel “dirty” or promiscuous.
  “*Ya nin epekto sa mga pakat na ento ba kayan ako amengka aden VDRL nengka. Na ya nila talon ka kna kapegkudi na mama endo maledsik kon e lugo ko*”.
  (The effect on my friends is that I am ashamed because I’m positive with VDRL. They said that one of the causes of my disease is having sexual intercourse with men and my blood is dirty.)
  This woman’s husband was also affected by the positive blood test of his wife. Even he felt shame and could not socialize even though he was not infected.
  “*Kalido e ginawa ko ka napa examine sa lugo na maledsik kon e lugo nin*.” (I pity her because when her blood was examined, they said that her blood was dirty.)
  “*Kayan ako. Dili kami paka-simbol sa kadakelan a taw.*”
  (I am ashamed. We can’t mingle with many people.)
  Yaws is also stigmatizing for adult males with positive screening tests because they are thought to have had sexual relations with promiscuous women or prostitutes. An adult male participant replied:
  “*Akala tuloy gumagamit ako ng maduming babae.*” (They think I use a “dirty’ woman.)
  One participant reported that the affected person would not have sexual contact because of a yaws infection.
  A government physician of Maguindanao had encountered young, unmarried, female patients in the past who had positive VDRL screening tests even without any high-risk behaviors or sexual contact. These women felt distraught and ashamed if others heard about their test results, because their honor as a single woman would become questionable. They would often wonder how they became infected or how their test result became positive.
  Pregnant women who were required to undergo prenatal screening using rapid tests for syphilis were also surprised and ashamed to have positive tests. The serologically positive patients would not usually know that yaws could also make their blood tests positive for life. Oftentimes, they would consult physicians to find out how they could have treatment and eventually have negative VDRL, RPR, or Treponemal antibody tests.
2. Negative effects on children
  Children with yaws were observed by adult participants to be bullied and ashamed. Playmates would tease the affected student and call them names like “*bakatawon*” (one who has yaws)*,* making him/her not want to go to school or mingle/interact with others.
  One adult who had yaws in childhood said:
  “*Tinutukso kaming batang bakataw*” (We children with yaws were teased).
3. *Depression, isolation, and shame* were also felt by yaws patients especially among the adults. They avoided social interactions and felt discrimination from others. They feared passing on the infection. Disappointment and sadness were pervading feelings, because the adults affected could not be employed overseas.

#### Economic effects of yaws

The negative economic effects of yaws mainly stem from the patient’s ineligibility to be employed as overseas workers once their screening tests were positive (VDRL/RPR/Treponemal antibodies). They were automatically considered “unfit for work” or “unfit for work abroad”.

A 42-year-old woman from Dadtumeg, Maguindanao viewed her positive VDRL test results as a hindrance to employment.

“*Kalido e ginawa ko ka pegkyug ako pagabroad na di kapakay sa medical ko.*” “*Sa buhay ko na pakaangga talaga.*” (I am disappointed because I want to go abroad or another country but it is not possible because of my medical record (positive with VDRL). It is really a hindrance to my life.)

Key informants described yaws as “income-depleting”. There were overseas workers who initially passed the laboratory screening test in the Philippines but were positive for Treponemal antibodies when retested abroad, resulting in their deportation. These individuals had already spent large amounts of money or sold their agricultural land, animals, or other property which were their only means of livelihood.

According to health workers interviewed, although yaws was fully treated in some patients, the Treponemal or non-treponemal antibodies remained reactive. They still could not be considered fit for work abroad. The lost opportunity created by past infections of yaws or latent infections has created a large problem of unemployability and lack of income for a lifetime among the communities where yaws exist.

“*Ya nin epekto sa pamilya na di silan katabangan sa kabamantiyale.*” (The effect in my family is that I can’t help in earning a living.), said our 42-year-old female participant.

Her husband had a bleak view of the future if his wife could not be treated and work abroad. “*Dabon amengka maya bon ba. Dala gamot, kalunsanan so ka miskin.*” (Nothing will happen if there is no medication for her.)

The direct cost of treating yaws also affected the finances of the patient. One health worker participant noted that patients do not have money to pay for treatments. Patients who were disturbed by the itching or wounds also could not work (ex. fishing).

The summary of the physical, psychosocial, and economic effects of yaws on those affected by yaws in the Southern Philippines are summarized in Table 2.

**Table 2:** Summary of physical, psychosocial, and economic effects of yaws

Physical	Psychosocial	Economic
• Skin manifestations with bone/cartilage deformity • Multiple scars make skin rough • Irritable because itchy • Disturbs work due to the itch • Bone pains	• Affects activities of daily living because of depression, isolation, discrimination of others • Disgusted and depressed • Embarrassed to go out • Ashamed due to (+) VDRL, “dirty” blood • Avoids others, afraid to infect others/be infected • Disappointed, because he cannot work abroad	• Income-depleting • Cannot earn a living (ex. could not go fishing)
• Normal activities of daily living in early stage, difficulty if late stage • Affects daily routine because of cleaning the wound	• Being bullied and ashamed • Playmates would tease affected students • Does not want to go to school • Does not want to mingle/interact with others	• Hinders overseas employment due to (+) serological tests during pre-employment screening
• Very degrading, repulsive because of the smell (fishy) and appearance of disease • Lesions are unsightly, unhygienic • “*Kadiri*” (disgusting)	• Stigma when applying for work abroad because one is thought to be positive for syphilis • Cannot work well because of stigma	• Pregnant patients with (+) RPR not able to work abroad • Unfit for work abroad • Unfit for employment
	• Suspected or considered promiscuous or sexually immoral (they think I use a “dirty’ woman) • No sexual contact	• No money to spend for treatment
	• *No effect* • *No stigma because of poor knowledge and little understanding from community*	• *Not applicable, because patient is in the pediatric age group* • *Not applicable, since patient is elderly*
	• *Still interacts with others*	• *Not observed in community*

Italicized: no effect

## Yaws II. Aetas of Quezon Province

In the second yaws study conducted in 2020, yaws was detected only among an indigenous people found in one out of the five study sites screened.

The Aetas residing in Sitio M, Sto. Tomas, Tagkawayan, Quezon belong to a group of semi-nomadic families that move back and forth from Camarines Norte (the adjacent province) and Quezon province. They reside at the foot of the Sierra Madre Mountain range. The number of individuals is estimated to be 59 in the year 2020. The Aetas have been panning for gold (referred to as “pagkabod”) in the river of Sitio M and this has been their main source of livelihood. They spend several hours immersed in the river water, and after gathering enough gold dust, they smelt it, and sell the gold.

### Personal knowledge, beliefs and attitudes including social stigma

#### Local disease terms for yaws

When asked what they call their skin disease, 7 Aetas referred to it as “*alipong*” or “*alipunga*”, the Tagalog term for tinea pedis, since they had plantar yaws. Two participants called the disease “*bakatuy*” which is similar to the term “*bakataw*” of Maguindanao.

This similarity may be due to the social preparation given by the study personnel prior to the survey when the term “*bakataw*” was used in information materials and orientations.

There was only one other study site that had residents who reported seeing yaws-like skin lesions in the past and in their communities. The local leaders of the Dumagat/Remontados of Rodriguez, Rizal were oriented on yaws and asked if they had ever seen such skin lesions before. Some of them had seen yaws-like lesions in the past and referred to yaws as “*bute*”.

One local health worker in the Basey, Samar study site remembered the yaws eradication campaign and had seen yaws-like skin lesions in the past. He reported that yaws was called “*kapithok*” in the Waray language.

#### Signs and symptoms

When asked about how the yaws lesions developed, 8 participants’ lesions initially affected the dorsa of their feet. Lesions started as wounds (*n* = 3), whitish (*n* = 3), or papules (*n* = 1) that increased in number, were recurrent, and spread around their feet. Pruritus or painful lesions were also reported. One participants felt numbness after walking all day. One participant could not recall how the skin lesions developed.

### Life events attributed to the disease: perceived causes of yaws

Eight participants thought that their skin disease was due to being immersed in the river water, while 5 of them specified that disease occurred with exposure to turbid or dirty water (“balinabon”). One participant explains that “balinabon” occurs when one stirs the river water and it becomes turbid or murky.

“*Pag lumulublob po sa tubig, kapag may balinabon po, doon po nakukuha*” (When one is immersed in the water, when there is turbidity, that is where one can get it)

Three participants mentioned that yaws developed due to gold panning (“*nakuha sa pagkakabod*”), since they had to be immersed in the river water often.

### Current behaviors and behavioral predispositions about managing the disease: health-seeking behaviors

All the participants had not sought treatment for their yaws lesions in the past, up until the study physician (a Rural Health Unit physician) approached them. Their local remedy for yaws lesions was diesel called “*krudo*” (*n* = 7) or a souring agent called “*pampaasim*” (*n* = 2). The household contact interviewed did not use any remedy for the yaws lesions of her husband.

### Socioeconomic effects of yaws

#### Feelings about the disease

Most participants felt well and did not feel their skin disease was a problem. The father of one of the active yaws patients (i.e., the 8-year-old boy with bowed forearms) was interviewed. He was also had past yaws. He was worried whenever the wounds would increase in number in his son.

#### Effect of yaws on relationships and view of themselves and the future

All the participants did not feel any effect of the skin disease on their relationships with members of the household and community members or on how they viewed themselves. Household contacts did not feel any effect or worry about having a yaws case in the household. The father of the active yaws patient did not observe any effects of his son's yaws on relationships with others, such as avoidance. He explained that yaws was nothing new in their community. Three past yaws participants explained that the skin disease was found in many of their community members anyway.

“*Hindi naman po nakakaapekto. Ganyan naman po karamihan dito.*” (It does not affect me. Most of us have it (yaws) anyway.

#### Economic effects

The yaws lesions lead to interruptions in their livelihood, gold panning. The recurrent painful or itchy wounds on the feet cause the participants to stop work as gold panners. They have to wait for the wounds to heal before they can resume work.

A 25-year-old male reported that he has to stop work (gold panning) when skin lesions become itchy and painful. He applies diesel to dry up the lesions.

“*Pag makati at masakit po ay minsan, tigil sa pagkakabod. Papatuyuin muna ng krudo.*” (When it is itchy or painful sometimes, I stop gold panning. I let it dry up first using diesel).

Table 3 summarizes the effects of yaws among the Aetas of Quezon.

**Table 3:** Summary of physical, psychosocial, and economic effects of yaws among Aetas of Quezon

Physical	Psychosocial	Economic
Pruritic or painful wounds and papules; whitish	*Most do not perceive the disease as a problem, because the disease is common among them*	Pain of the wounds on the feet makes them stop work (gold panning) in the river
Initially affects the feet, creating wounds on the soles	*No effect on social relationships*	
Recurrent	A parent worried about the recurrent wounds in the child	
No treatment or medical consultation sought in the past		

Italicized: no effect

## Discussion

The two yaws case detection studies of 2017 and 2020 confirmed the presence of active, latent, and past yaws in the Luzon and Mindanao island groups of the Philippines. Although there is a possibility of treated or latent syphilis infection among adults and older adolescents that only polymerase chain reaction (PCR) could distinguish, the diagnosis of latent or past yaws is more likely due to the historical and current presence of yaws in the area [27], the low prevalence of syphilis [28], and the low-risk profile for sexually transmitted infections of these affected individuals living in remote villages.

In these studies, we found that yaws has significant negative physical, psychosocial, and economic effects especially in the Mindanao, Southern Philippines. The malodorous, moist, cauliflower or yellow-crusted papillomas and wounds are considered unsightly or repulsive and tedious to clean and treat. Activities of daily living are hindered. Stigma, discrimination, and embarrassment are common. Children get bullied and miss school days to avoid bullying. Social interaction and physical intimacy are avoided due to fear of spreading the infection. Employment opportunities are less when the adult is positive for screening blood tests such as RPR or VDRL or for treponemal antibodies. Even if they do not have active yaws, those who have latent yaws (i.e., serologically positive for treponemal and non-treponemal tests) or past yaws (i.e., serologically positive for Treponemal antibodies) cannot be accepted for employment abroad.

Health-seeking behaviors of yaws patients or their parents tended to be self-medication or neglecting the skin lesions until they became worse. Lack of accessible health centers and medications were also cited as reasons for not consulting early. Treatments are not readily available for the populations affected thus perpetuating the infection and negative effects. We found one cross-sectional survey on the knowledge, attitudes, and practices among yaws patients in Ghana but it did not include stigma or psychosocial effects [29]. Our study found perceptions of yaws that were similar to the findings in Ghana such as the belief that yaws was contagious and due to dirty water or poor personal hygiene; however, our participants did not attribute yaws to supernatural forces unlike some participants in Ghana.

While results of the cross-sectional surveys provide generalizable health information, the ethnographic method using rapid assessment procedures was used to gain deeper insights into the personal images, meanings, motivations and decision-making processes of risk-prone individuals and those with confirmed yaws. The in-depth interviews sought to analyze the nature and dynamics of social stigma as related to yaws. The outputs of the exploratory phase included local terminologies for yaws, community cognitions and attitudes toward the disease, and local practices related to its prevention and treatment. These interviews provided vital information about potential barriers to the early detection and treatment of yaws or potential enabling factors for a successful yaws eradication program.

### A comparison of the effects of yaws among Filipinos

Yaws was found to have different effects on the two groups of Filipinos studied: physically, psychosocially, and economically.

1. The Aetas of Quezon reported more of physical discomforts from the painful plantar yaws wounds rather than psychosocial problems. The painful wounds had economic consequences, because the Aetas had to stop work in the river and could not earn money from gold panning. Social relationships were not impaired because yaws was commonplace among the Aetas and not associated with promiscuity.
  These findings contrast with those from the 2017 study in Mindanao, Southern Philippines. Yaws skin lesions also caused physical discomfort and pain. However, of more importance were the generated stigma and discrimination among yaws-affected residents of Mindanao, because positive serological tests for Treponemal or non-Treponemal antibodies were associated with sexually transmitted infections such as syphilis and with promiscuity.
  In Mindanao sites, children with active skin lesions were often teased, whereas there were no reports of teasing among Aeta children. In a study conducted in West Africa, a modified Children’s Dermatology Life Quality Index Questionnaire was used and Focused Group Discussions were conducted to investigate the impact of common skin diseases on school children. Social relationships were often affected, wherein children having skin diseases were mocked, rejected or isolated causing affective changes as well as behavioral changes, such as isolation, self-restraint and self-stigma [30].
2. Our study revealed that the economic effect of yaws was another obvious difference between the two groups. The Aetas did not report problems with employment, since their livelihood was mainly gold panning and not overseas work. The Mindanao participants with latent or past yaws infection were adversely affected in terms of opportunities to work abroad because applicants with reactive non-Treponemal or Treponemal antibody tests could not be eligible.
  Weiss pointed out that “someone excluded from a job, because they have a condition, even though that condition does not prevent them from fulfilling the requirements of the position, is in a different category with regard to social stigma from someone who is excluded (removed or not hired), because they cannot fulfill the requirements of the position” [3]. The adults with past yaws who have been applying for overseas work have been experiencing this form of social stigma for several years and expressed sadness because their only health issue was a positive screening test for syphilis and there was no remedy for this.
3. Language reflects culture, shared experiences, and the importance given to a disease. The participants from Southern Philippines immediately knew the specific local terms for yaws (*bakataw*, *pamali*) and their corresponding morphologies, reflecting the long-standing presence of the disease in the area. However, the Aetas of the northern region of the Philippines did not have a specific disease term for yaws, referring to the plantar wounds as “alipong” or “alipunga” which are local terms for tinea pedis, a fungal infection of the feet. The plantar wounds were the most disturbing effects of yaws for them. The Aetas did not have a specific term for the yellow-crusted, papillomatous skin lesions nor the bony deformities associated with yaws. Yaws had been endemic in their community for several years and they considered these skin lesions as commonplace but not socioeconomically disturbing.
  A search for historical records of the local terms for yaws revealed that the Visayan term for yaws are “puku” and “tabukaw” [25]. During the Spanish colonial era, yaws was referred to as “bubas” [26].

### Yaws, syphilis, and stigma

Yaws and syphilis share the same causative agent—*Treponema pallidum*. Both cause chronic infections which manifest as single lesions, progress to disseminated lesions, and ultimately may cause destruction of tissues including skin, cartilage, and bone. They differ in the mode of transmission, where syphilis is transmitted sexually and yaws through skin contact [31]. Serological assays remain the most common diagnostic method for all treponemal infections. Importantly, none of the currently available assays can differentiate between infection with syphilis and infection with any of the endemic treponematoses [32–34]. The stigma of syphilis often accompanies a positive screening test, since syphilis is historically associated with prostitution and promiscuity [35–37].

How did yaws become stigmatizing in Southern Philippines? Adults applying for work abroad are required to have pre-employment tests for sexually transmitted infections (STIs), including syphilis [38] especially in Middle East Countries [39]. If they test positive for Rapid Plasma Reagin (RPR), a confirmatory test is recommended. Upon arrival in the Middle Eastern country, the screening for major STIs (i.e., syphilis, HIV) is repeated within 4 weeks before a work permit can be issued. Workers diagnosed to have syphilis are given appropriate treatment but are sent back to the country of origin [40].

Syphilis is among the communicable diseases of public health importance that immigration authorities have to look out for to prevent its spread [41]. The pre-employment requirement of syphilis screening is motivated by public health considerations rather than the stigma associated with promiscuity. For this reason, work applicants are ineligible for overseas employment based on positive Treponemal antibody tests even though they could be non-infectious yaws (past or treated yaws).

Yaws became a stigmatizing disease among Filipino adults in Mindanao, Southern Philippines because of another public health measure. Syphilis screening is recommended in pregnant women [34, 42] because of the health risks of syphilis to the fetus. However, in yaws-endemic areas, such as Mindanao, pregnant women who underwent screening for syphilis may have experienced social stigma after testing positive. In 2009, Médecins Sans Frontières reported that 25.5 percent of pregnant women who were displaced due to armed conflict in Mindanao had tested positive for treponemal screening tests. The local health officer surmised that yaws could be the reason for the high number of positive tests [43]. These women were likely to have had latent or past yaws; however, clinical and serological confirmation of yaws were not yet conducted at that time.

In 1957, Gentle and Harold reported a similar situation as in our Mindanao study sites. Several people of all ages in Trinidad and Tobago had positive serological findings attributed to latent yaws and they were stigmatized due to the association with syphilis. Consequently, those affected by latent yaws had unnecessary expenses for consulting different doctors and for treatments. Our study also had yaws participants who expressed the desire to be treated to attain negative Treponemal tests and, just as reported by Gentle, they also felt “despair when they realised that for all their trouble their blood still remains positive.” [44].

Information dissemination and assessment of STI, such as syphilis, is attached to the Philippine HIV control program [45, 46]. This may be a factor in the stigma related to these diseases and to anyone who tests positive serologically for syphilis markers.

### Yaws affects all stages of life

**Fig. 3:**
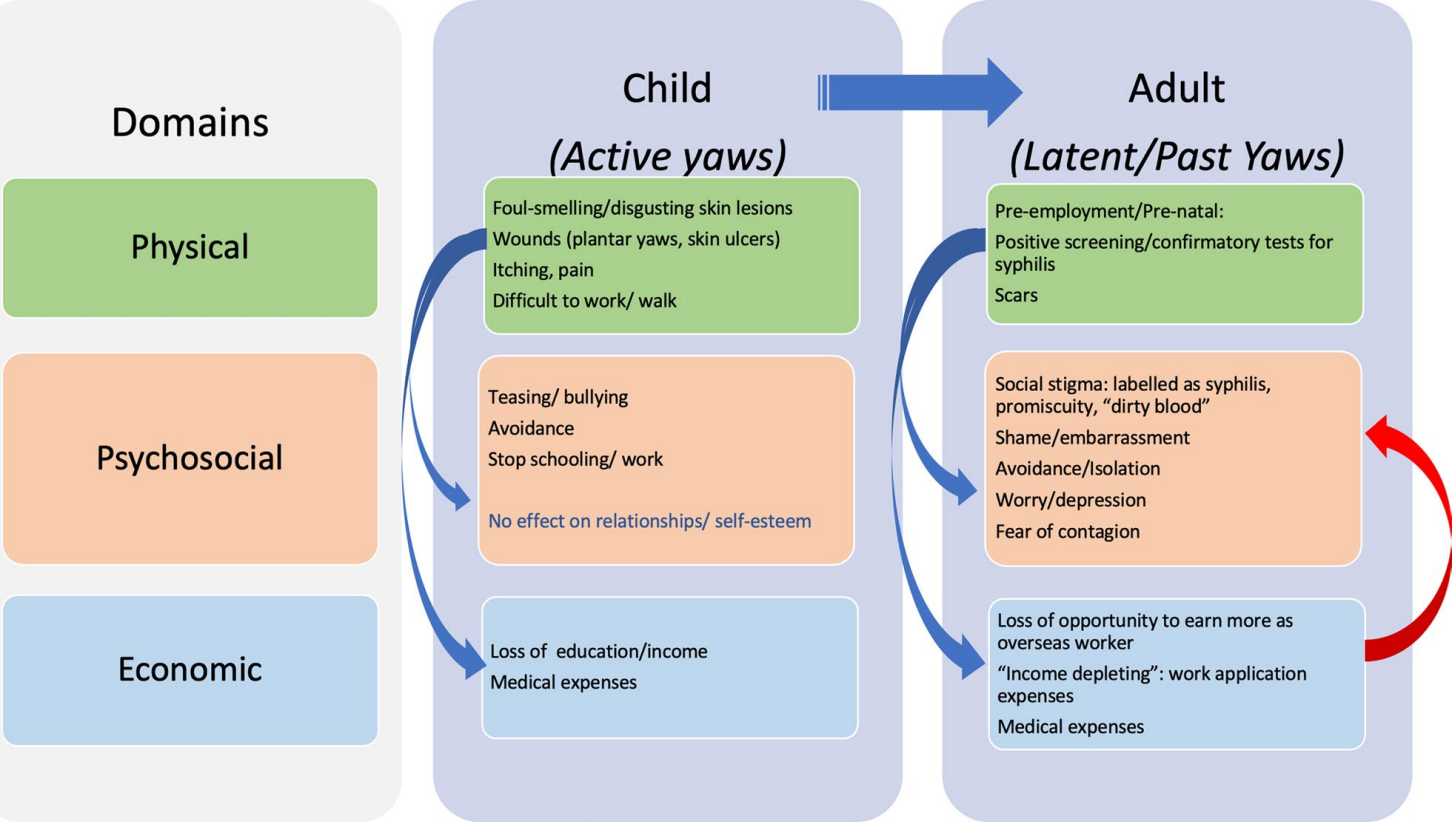
Physical, psychosocial, and economic effects of yaws from childhood to adulthood among Filipinos

Yaws is not just a physical problem during childhood but could lead to a lifetime of stigma and poverty. Based on our studies’ findings, we created a diagram to summarize the effects of yaws and the interaction of factors influencing the effects of yaws on an affected person’s life during childhood and into adulthood (Fig. 3). Yaws starts as a physical problem during childhood. Among children, yaws is mainly a physical concern due to the skin or bone lesions but with some negative psychosocial consequences or none at all depending on the culture. There is no escalation of the psychosocial problems, since skin lesions eventually resolve on their own. As yaws enters the latent phase in older childhood and adulthood, the persistent treponemal and non-treponemal antibodies become a lifetime problem, because these disqualify work applicants from overseas employment. Psychosocial problems, social stigma, and economic losses are more prominent during adulthood. Negative economic effects perpetuate the psychosocial problems among adults.

### Interventions to mitigate stigma related to yaws and other NTDs

The World Health Organization recommends an integrated approach to reduce the suffering, psychosocial impact, and stigmatization of skin NTDs, such as yaws [47]. Weiss and Ramakrishna proposed a framework that indicates the focus and approach for interventions to reduce stigma related to NTDs [48].

First priority is the proper control of the public health problem, such as yaws, early recognition, and treatment to cure and prevent disability. There should be available diagnostic tests (rapid point of care tests for *Treponema pallidum* and Dual Pathway Platform) and treatment (azithromycin or penicillin), early detection, surveillance, and prompt management in the locality or grassroots level, such as the village health station or local health center. Other possible endemic areas should be checked for yaws, and surveillance and case finding activities should be intensified with the help of teledermatology. Sufficient water and improved personal hygiene should be implemented especially in remote and impoverished communities where yaws may exist.

To reduce stigmatizers, information–education–communication (IEC) materials and social marketing are needed. There should be yaws training for the health sector and a widespread information campaign for the general public about the non-venereal nature of yaws and the potential eradication of yaws with mass drug administration. The public awareness about yaws can accomplish the following: (1) challenge cultural ideas that blame “victims”, i.e., misconception that a positive screening test could only be due to syphilis and promiscuous behavior, (2) provide alternative explanations for the positive screening tests for syphilis, i.e., yaws acquired in childhood, latent yaws, and (3) correct the unfounded concerns about the risks of people affected by yaws [3].

To mitigate the emotional impact of stigma, Weiss suggested counselling, peer support groups, and therapeutic communities for mental health problems. For example, our study participants felt relieved when we reassured them that non-venereal yaws was more likely than syphilis as a cause for the positive serologic tests and that it was a treatable infection.

How can we address the problem of ineligible job applicants because of their persistent Treponemal antibodies despite the treatment of active or latent yaws? Advocacy, lobbying, and legislation can improve social and public health policies. Perhaps immigration authorities and overseas employers can change their policies and consider work applicants who are diagnosed as non-infectious past or treated yaws if they had resided in known yaws endemic areas. Additional health social science research will increase our understanding of cultural meanings, guide health policy, and also include people affected by yaws in appropriate approaches and interventions.

### Limitations

We were not able to interview all participants with yaws in both study areas owing to their lack of availability or remoteness of their dwellings. Some of the responses on the participants' knowledge of yaws may have been influenced by the social preparation phase of the studies. Despite the exploratory nature of this study and the small number of participants, we were able to reveal a new and important dimension of yaws: the negative psychosocial and economic effects among Filipinos in endemic villages.

## Conclusions

Yaws is not merely a chronic skin and bone disease. This study’s findings indicate that yaws can lead to significant negative socioeconomic effects in communities where overseas employment is important, as in the Southern Philippines, and where intact skin is essential for work productivity, as with the gold panners among the Aetas of Luzon.

The Philippines does not have an existing yaws control program and there is a general lack of awareness about yaws. This means that yaws information and treatments are not readily available for the populations affected, thus perpetuating the infection and social stigma. This study’s findings can guide yaws control program planners on the socioeconomic and behavioral factors influencing the disease, the Information–Education–Communication materials to develop, and the approaches to the community for yaws control and eradication measures.

The reduction of stigma and discrimination due to yaws is a challenge. If the physical and psychosocial effects of yaws are addressed adequately and with sensitivity, we will have eradicated, not only yaws as an infection, but also its lifelong problems of stigma as well.

## Data Availability

The data that support the findings of this study are available from Philippine Department of Health’s Health Systems Research Management but restrictions apply to the availability of these data, which were used under license for the current studies, and so are not publicly available. Data are, however, available with permission of the Philippine Department of Health’s Health Systems Research Management. Email: aheadhpsr@doh.gov.ph.

## Abbreviations

NTD: Neglected Tropical Diseases
PCHRD: Philippine Council for Health Research and Development
SCMC–AEIERC: St. Cabrini Medical Center–Asian Eye Institute Ethics Review Committee
SJREB: Single Joint Research Ethics Board
UPMREB: University of the Philippines Manila Research Ethics Board
VDRL: Venereal Disease Research Laboratory
RPR: Rapid Plasma Reagin
STI: Sexually transmitted infection
